# RXR controlled regulatory networks identified in mouse brain counteract deleterious effects of Aβ oligomers

**DOI:** 10.1038/srep24048

**Published:** 2016-04-07

**Authors:** Kyong Nyon Nam, Anais Mounier, Nicholas F. Fitz, Cody Wolfe, Jonathan Schug, Iliya Lefterov, Radosveta Koldamova

**Affiliations:** 1 Department of Environmental & Occupational Health, University of Pittsburgh, Pittsburgh, PA, 15219, USA; 2 Department of Genetics, Perelman School of Medicine, University of Pennsylvania, Philadelphia, PA, 19104, USA

## Abstract

Bexarotene, a selective agonist for Retinoid X receptors (RXR) improves cognitive deficits and amyloid-β (Aβ) clearance in mice. Here we examine if the effect of bexarotene on RXR cistrome and transcriptomes depend on APOE isoform and Aβ deposition. We found bexarotene increased RXR binding to promoter regions in cortex of APOE3 mice. Rho family GTPases and Wnt signaling pathway were highly enriched in ChIP-seq and RNA-seq datasets and members of those pathways - *Lrp1, Lrp5, Sfrp5* and *Sema3f* were validated. The effect of APOE isoform was compared in APOE3 and APOE4 mice and we found significant overlapping in affected pathways. ChIP-seq using mouse embryonic stem cells and enrichment levels of histone marks H3K4me3 and H3K27me3 revealed that, bexarotene induced epigenetic changes, consistent with increased neuronal differentiation and in correlation with changes in transcription. Comparison of transcriptome in APOE3 and APP/APOE3 mice revealed that amyloid deposition significantly affects the response to bexarotene. In primary neurons, bexarotene ameliorated the damaged dendrite complexity and loss of neurites caused by Aβ_42_. Finally, we show that the disruption of actin cytoskeleton induced by Aβ_42_ *in vitro* was inhibited by bexarotene treatment. Our results suggest a mechanism to establish RXR therapeutic targets with significance in neurodegeneration.

Retinoid X receptors (RXRs) are ligand-activated transcription factors that heterodimerize with other nuclear receptors to generate functional transcription factors^1^. In brain, RXRs partner mainly with Liver X Receptors (LXRs), Peroxisome Proliferator-Activated Receptors (PPARs) and Retinoic Acid Receptors (RARs). RXR/LXR and RXR/PPAR pairs are considered permissive heterodimers because they can be activated by ligands of either receptor while RXR/RAR are non-permissive^1^.

Alzheimer’s disease (AD) is a late onset dementia characterized by extracellular deposits of amyloid-β (Aβ), intracellular aggregates of tau and cognitive decline. The major genetic risk factor is the inheritance of *APOEε4* allele of *APOE* gene which incidentally is RXR/LXR target gene. It has been repeatedly demonstrated, at least in experimental models, that RXR/LXR heterodimers act to regulate genes that are critically involved in AD pathogenesis (reviewed by Skerrett *et al*.^2^). Therefore, the therapeutic approaches aiming at pharmacological modulation of RXR/LXR activity, and thus changes in expression level of their target genes, have been explored for more than a decade now^3^,^4^,^5^,^6^. Bexarotene is a selective RXR agonists, FDA (US Food and Drug Administration) approved for treatment of Cutaneous T Cell Lymphoma^7^. Several studies have demonstrated that the application of bexarotene in AD mouse models improves cognitive impairment, including in mice expressing human APOE isoforms^6^,^8^,^9^,^10^. Regardless that the initially reported effect on amyloid plaques^6^ was not confirmed^9^,^10^,^11^,^12^,^13^ several studies demonstrated a decrease of soluble Aβ in the brain^8^,^12^,^14^ and one study demonstrated that the effect depends on *Abca1*^15^. Importantly, we and others have demonstrated that bexarotene treatment decreased Aβ level in brain interstitial fluid of APP transgenic mice^6^,^9^. A recent double-blind, placebo-controlled clinical trial showed that bexarotene reduced brain amyloid and increased serum Aβ42 in *APOEε4* non-carriers but demonstrated an increase of serum triglycerides in bexarotene-treated patients^16^. Interestingly, Omega-3 Fatty Acids were shown to augment the effect of bexarotene on amyloid load and at the same time to decrease bexarotene-induced hypertriglyceridemia in AD model mice^17^. The effects of Targretin^®^ (bexarotene) on cognition and biomarkers were recently reported in a patient with mild Alzheimer’s disease (AD). Remarkably, after 6 months of treatment at 300 mg/day, memory improved by about 40% and the level of tau in CSF decreased by about 20%. No significant side effects were recorded^18^. Bexarotene was tested in mouse models for other neurodegenerative disorders, such as Parkinson disease^19^ and Amyotrophic Lateral Sclerosis^20^ with promising results. Results of two clinical trials conducted with patients with schizophrenia or schizoaffective disorders have been published and demonstrated reduction of scores recorded on Positive Syndrome Scale (PANSS) in bexarotene treated patients, compared to those in placebo treated^21^. Recently, we have reported bexarotene controlled genome-wide networks of neuronal differentiation and neuritogenesis in mice expressing human APOE4, as well as increased number of neuronal progenitors in the dentate gyrus of APOE3 and APOE4 mice after bexarotene treatment^22^. Data from our and other groups have demonstrated that in APP transgenic mice bexarotene affects inflammatory and immune response in the brain, the expression of genes involved in Aβ phagocytosis by microglia, such as *Trem2, Tyrobp, Apoe, Mertk,* as well as Aβ phagocytosis *in vivo* and *in vitro*^23^,^24^, and the phagocytosis of myelin debris which in turn affects the remyelination^25^, suggesting that complex networks are affected by RXR activation with possible implication for neurodegenerative disorders.

The aim of this study was to determine if the effects of bexarotene depend on APOE isoform or Aβ deposition. We applied high-throughput sequencing to map RXR cistrome and to reveal changes of the transcriptome in APOE3 mice treated with bexarotene. To determine the effect of APOE isoform, we compared the results from APOE3 mice with published data from APOE4 mice and identified the common Gene Ontology categories that are affected by bexarotene in both genotypes. To identify the effect of amyloid deposition on transcriptome, we performed RNA-seq using brain tissue from APP/E3 mice and tested how bexarotene counteracts oligomeric Aβ toxicity.

## Results

### Bexarotene affects the distribution of RXR binding to genomic sites in mouse brain

**Figure 1:**
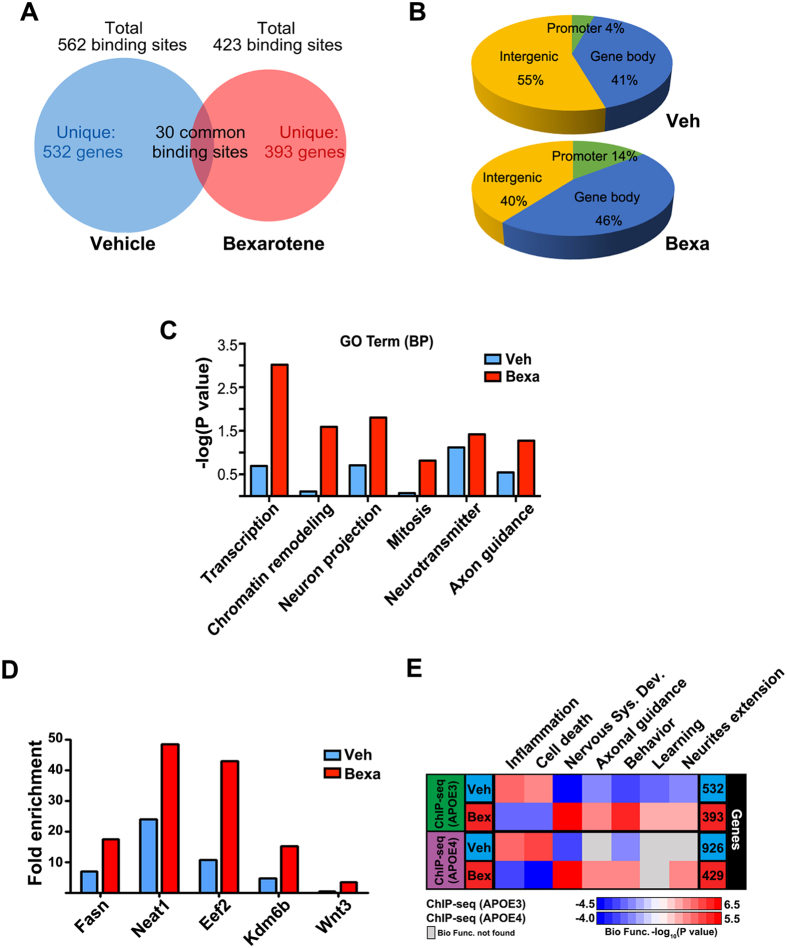
ChIP-seq data reveal changes in the distribution of RXR binding in APOE3 mice brain induced by bexarotene treatment. APOE3 mice were treated with bexarotene or vehicle as described in Materials & Methods and ChIP-seq was performed using material from cortex. (**A**) Venn diagram showing the number of overlapping peaks in bexarotene (Bexa) and vehicle (Veh) treated APOE3 mice (Blue: Veh, Red: Bexa); (**B**) Distribution of RXR binding showing significant shift towards RXR binding in promoter regions of annotated genes in bexarotene versus vehicle treated mice. (**C**) Most significant functional annotation categories (GO Term Biological Process, DAVID); (**D**) ChIP-qPCR validation data of genes with enriched RXR binding in promoter regions. *Fasn* gene is used as a positive control. (**E**) Comparative heatmap of RXR cistrome of bexarotene treated APOE3 and APOE4 mice Shown are the most significant distinct Biological Function categories identified by IPA in each experiment. Blue represents vehicle and red represents bexarotene. Gray boxes indicate no overlapping categories. Negative log_10_ (P value) denotes lower significance and red color higher significance.

In our recent study we demonstrated bexarotene increased RXR binding to genomic sites associated with genes involved in neuronal differentiation and neuronal projections in APOE4 mice^22^. To determine if RXR binding is affected by APOE isoform we applied chromatin immunoprecipitation followed by high-throughput sequencing - ChIP-seq, using cortices of bexarotene treated APOE3 mice. The regions of enrichment in RXR datasets were identified at False Discovery Rate (FDR) < 0.01. RXR binding sites were connected to 562 genes in vehicle treated mice and 423 genes in bexarotene treated mice (Fig. 1A). Importantly, even though the number of total binding sites was slightly decreased by bexarotene, the treatment caused a significant increase in RXR binding to promoters. As shown on Fig. 1B there was a significant shift towards RXR binding in promoter regions of annotated genes in bexarotene treated, compared to control mice (10% vs 0.4%, Fig. 1B). In contrast, RXR binding to intergenic regions was significantly decreased (40% in bexarotene vs 55% in control). Functional annotation clustering using DAVID^26^ revealed that Gene Ontologies (GO) Biological Process (BP) “transcription”; “chromatin remodeling”, “neuron projections” and “axon guidance” were significantly increased in bexarotene treated mice (Fig. 1C) in agreement with our published data for APOE4 mice^22^. To validate the ChIP-seq datasets, we performed ChIP-qPCR for selected genes with enriched RXR binding using cortices of bexarotene and vehicle treated APOE3 mice (Fig. 1D). As shown, *Neat1* (Nuclear paraspeckle assembly transcript 1), *Eef2* (Eukaryotic elongation factor 2), *Kdm6b* (H3K27 demethylase 6b) and *Wnt3* (wingless-type MMTV integration site family, member 3) had an increased RXR binding in Bexarotene treated samples. We used IPA web tool, as previously^22^ to compare the biological function categories affected by bexarotene in APOE3 and APOE4 mice. As shown on Fig. 1E, the comparison of those categories identified by ChIP-seq showed that several pathways related to “neuronal development”, “neurite extensions” and “axonal guidance” were highly enriched by bexarotene treatment in both APOE3 and APOE4 mice. These results demonstrate there is a common signature of Genome-wide changes in RXR cistrome in response to bexarotene in APOE3 and APOE4 mice.

### Bexarotene significantly affects expression of genes related to nervous system development and neurites extension

**Figure 2:**
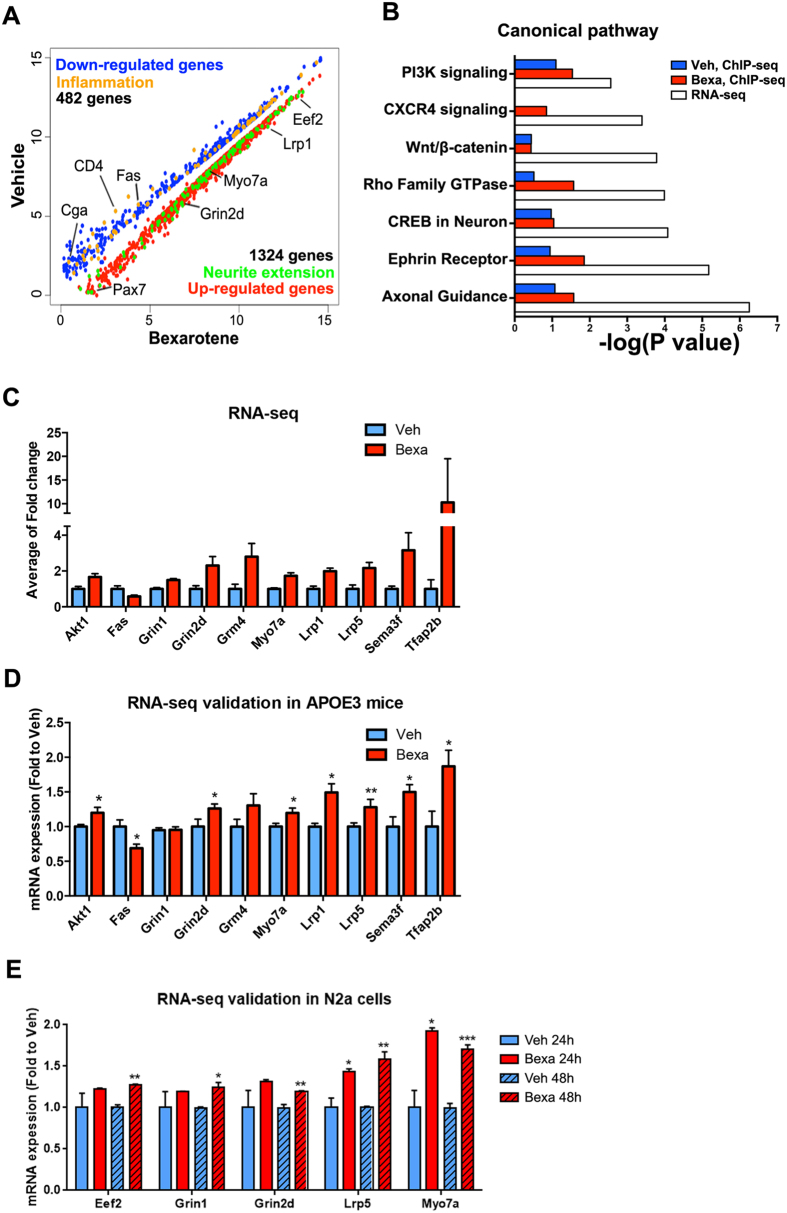
Bexarotene treatment significantly affects genes related to nervous system development, axon guidance and neurites extension. RNA was isolated from cortices of the same APOE3 mice treated with bexarotene or vehicle as shown on Fig. 1. (**A**) Scatter plot of transcripts in vehicle and bexarotene treated APOE3 mice. Transcripts identified as significantly differentially expressed at p < 0.05 cut-off are colored in blue (down-regulated) and red (up-regulated). Up-regulated transcripts associated with neurite extension are shown in green, and down-regulated genes associated with inflammation in orange. Gene symbols of representative transcripts are shown next to the corresponding values. (**B**) Most significant common canonical pathways identified by ChIP-seq and RNA-seq in APOE3 mice; (**C**) Significantly changed levels of RNA expression of genes in categories “neuron differentiation” and “neuron projection”; statistics is by edgeR, p < 0.05, n = 4/group; (**D**) Validation of RNA-seq results by qPCR; APOE3 mice, n = 4/group; (**E**) Validation by qPCR in N2a cells: Cells were treated with 1 μM bexarotene or vehicle for 24 hours and 48 hours (details in Materials & Methods). Values are mean ± SEM. For (**D**,**E**) statistics is by *t*-test; n = 3, *p < 0.05, **p < 0.01, ***p <  0.001.

Next, to determine how bexarotene affects mRNA levels in brain, we applied high-throughput RNA sequencing with RNA isolated from cortices of the same mice used for ChIP-seq. We found 1324 up-regulated genes and 482 down-regulated genes at P < 0.05 as a cutoff (Fig. 2A). Using IPA, we identified the most significant categories affected by bexarotene. As shown on Fig. 2B, the comparison of canonical pathways identified by ChIP-seq and RNA-seq revealed several pathways related to neuronal development and differentiation that were highly enriched in both data sets derived from bexarotene treated APOE3 mice, such as Axonal guidance, Rho family GTPase, Wnt and Ephrin receptor signaling, CREB signaling and CXCR4 pathway. To validate RNA-seq data, we selected genes that are differentially affected (cut-off p < 0.05) as identified by the canonical pathways shown on Fig. 2B. Specifically we chose genes that can affect neuron development (NMDA receptors *Grin1* and *Grin2d*^27^, *Grm4*, and *Myo7a*), neurite complexity (*Lrp5, Sema3f* and *Akt1*), neuronal death (*Fas*) or are related to Aβ (*Lrp1* and *Tfap2b*). As shown on Fig. 2D,E, the effect of bexarotene on *Grin2d, Lrp1, Lrp5, Sema3f, Tfap2b*^28^) and *Fas* as identified in RNA-seq data (Fig. 2C) was validated by qPCR in cortices of APOE3 mice (Fig. 2D) and N2a cells (Fig. 2E).

### Bexarotene induces epigenetic changes in ES cells consistent with increased neuronal differentiation

**Figure 3:**
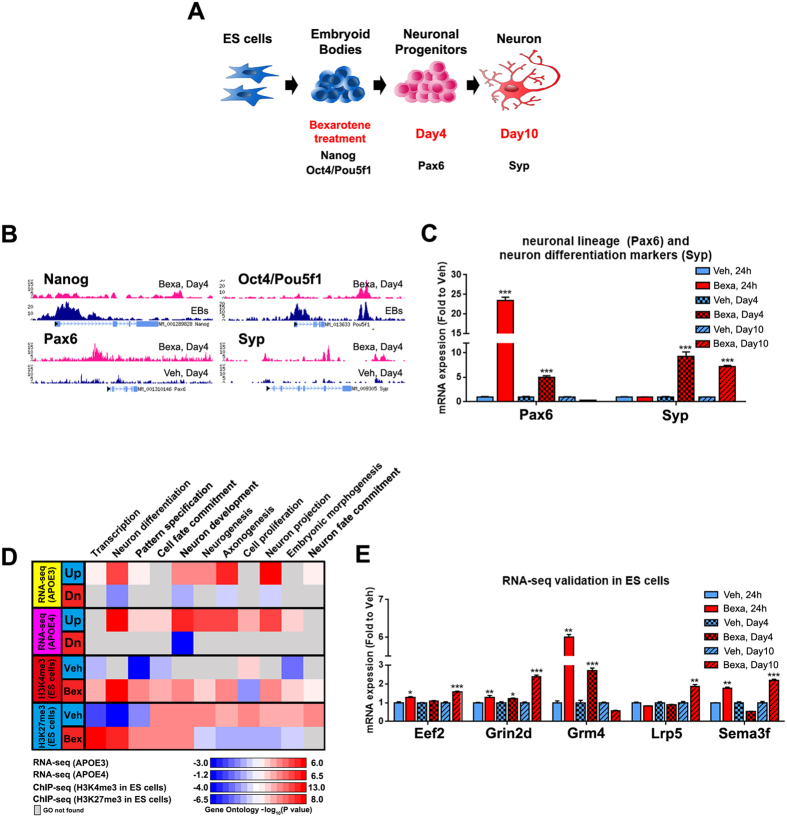
Bexarotene induces epigenetic changes in correlation with neuronal differentiation in ES cells. ES cells were treated with 5 μM Bexarotene for 4 days and markers for neuronal differentiation were examined 6 days after plating (Day10, Neurons). Control cells were treated with vehicle and processed in the same way. (**A**) Outline of the protocol for ES cell treatment and differentiation. EBs, embryoid Bodies; (**B**) Genome browser view of lineage markers in ES cells for H3K4me3 identified by ChIP-seq at EBs stage or 4 days after bexarotene (Bexa, Day4) and vehicle (Veh, Day4) treatment. Note that for pluripotency markers *Nanog* and *Oct4/Pou5f1* data are compared at EBs vs Bexa, Day4, and neuronal markers *Pax6* and *Syp* are compared 4 days after bexarotene or vehicle treatment. (**C**) Bexarotene treatment increases the expression of *Pax6* and *Syp*; (**D**) Comparative heatmap of significantly changed GO categories (gene lists derived from edgeR output tables) in APOE3 and APOE4 mice and epigenetic changes (analyzed ChIP-seq datasets) in bexarotene or vehicle treated ES cells (Day 4). Shown are the most significant distinct BP categories as determined by David in each experiment. Blue color indicates up-regulated genes and red indicates down-regulated genes in RNA-seq. For ChIP-seq data, Blue represents vehicle and red represents bexarotene. Gray boxes indicate no overlapping categories. Negative log_10_ (P value) denotes lower significance and red color higher significance; (**E**) Validation of RNA-seq results by qPCR in ES cells; Cells were treated with 5 μM bexarotene or vehicle for different time (24 h, Day 4 and Day 10). Values are mean ± SEM and statistics is by *t*-test; n = 3, *p < 0.05, **p < 0.01, ***p < 0.001.

As shown on Figs 1 and 2 and in agreement with the data from our previous study^22^, neuronal development and differentiation are categories significantly affected by bexarotene. To determine if bexarotene induces epigenetic changes consistent with differentiation, we performed ChIP-seq using ES cells to map the enrichment of two histone marks - H3K4me3, associated with active promoters, and H3K27me3 - a strong mark for repressed promoters, and also, associated with Polycomb repressive complex. We treated ES cells with bexarotene at the stage of embryoid bodies (EBs) for 4 days (experimental design shown on Fig. 3A) followed by ChIP-seq for H3K4me3 and H3K27me3 enrichment. As seen on Fig. 3B, the enrichment of H3K4me3 mark in promoters of pluripotency-associated genes, such as *Oct4* (alternative name *Pou5f1*) and *Nanog,* was significantly decreased 4 days after bexarotene treatment compared to the expression at the start (a stage of embryoid bodies). These data are consistent with the expression of these genes only in the pluripotent progenitors and their down-regulation in cells committed to neuronal lineage^29^. In contrast, the enrichment of H3K4me3 mark in bexarotene treated cells was increased in the promoters of *Pax6* (a marker for neuronal progenitors) and *Syp* (Synaptophysin) which is a marker for differentiating neurons. As seen from Fig. 3C, mRNA expression of *Pax6* was increased 24 hours after bexarotene treatment, corresponding to its role in the transition of ES cells to the neuronal lineage. *Syp* mRNA was increased at Day4 after treatment as in our previous study^22^. The results demonstrate that the expression of *Pax6* and *Syp* correlated to the enrichment of H3K4me3 at their promoters.

Next, we correlated the enrichment of H3K4me3 and H3K27me3 datasets in each gene ontology (GO) category to the gene expression data obtained from APOE3 and APOE4 mice treated with bexarotene. The heat-map on Fig. 3D shows that in both, APOE3 and APOE4 mice, GO categories “neuron differentiation”, “axonogenesis” and “neuron projection” are highly enriched in the bexarotene treated group. Importantly, the expression data highly correlated to the enrichment of H3K4me3 mark observed only in bexarotene treated ES cells. In agreement with these data, the enrichment of H3K27me3 mark was significantly changed between bexarotene and vehicle treated ES cells in categories such as “neurogenesis”, “neuron projection”, “axonogenesis” and “neuron fate commitment”. To validate these results, we examined mRNA expression level of RXR targets with relevance to neuron development and neurite extensions. As visible from Fig. 3E, *Eef2, Grin2, Sema3f* and *Lrp5* were increased 10 days after treatment corresponding to the stage of immature neurons. In contrast, *Grm4* was increased 24 hour after bexarotene treatment, suggesting an early response to bexarotene. Collectively, these data indicate that bexarotene-activated RXRs affect epigenomic landscape, consistent with the transition of ES cells towards neuronal cells.

### Aβ deposition alters bexarotene effects on the transcriptome in APP/E3 mice

**Figure 4:**
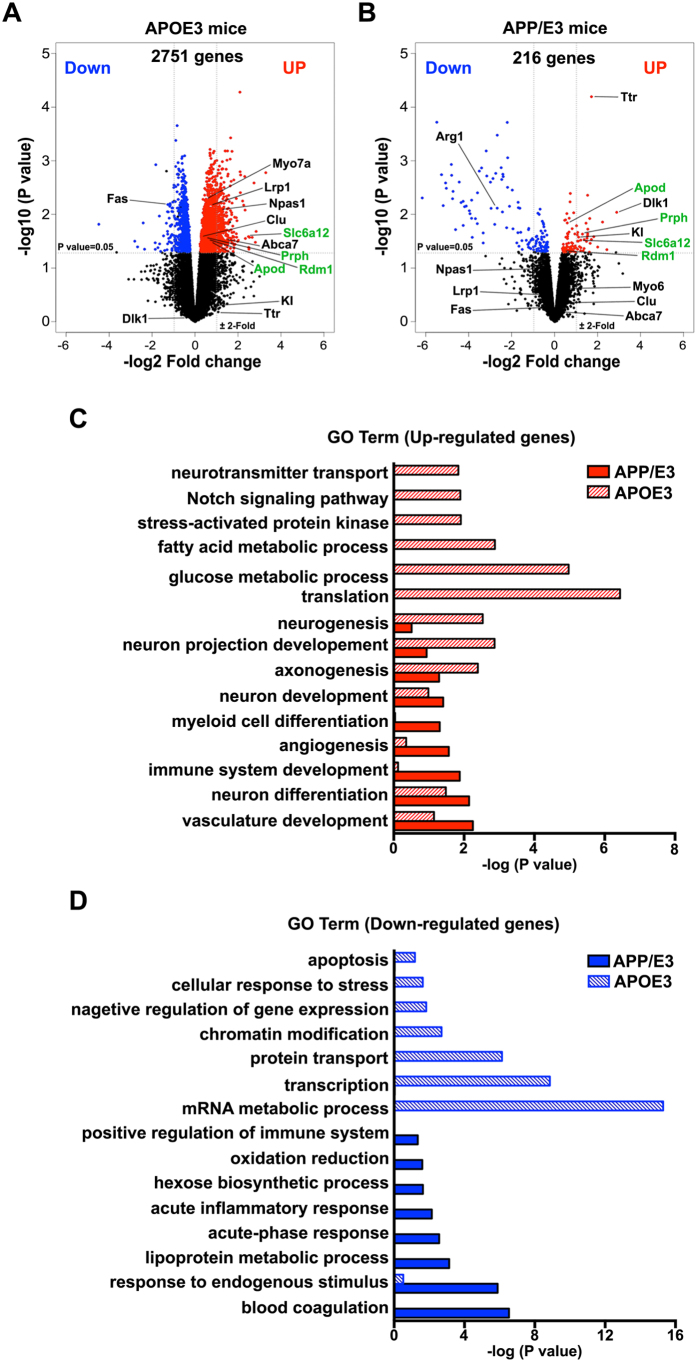
Aβ deposition differentially affects transcriptome changes in response to bexarotene in APOE3 and APP/E3 mice. Six month old mice of both APOE3 and APP/E3 genotypes were treated with bexarotene or vehicle (n = 4 for each condition) and RNA-seq datasets were used to determine differential gene expression. (**A**,**B**) Volcano plots for comparing transcripts in vehicle and bexarotene treated APOE3 and APP/E3 mice. Transcripts identified as significantly differentially expressed at P < 0.05 are colored in blue (down-regulated) and red (up-regulated). Gene symbols of representative genes associated with Aβ clearance and phagocytosis are indicated for both genotypes. The gene symbols in green denote common genes in both APOE3 and APP/E3 mice. (**C**,**D**) Comparison of the most significant distinct functional categories (GO Term Biological process) of differentially affected genes in both APOE3 and APP/E3 mice as identified by DAVID. (**C**) Up-regulated genes and (**D**) Down-regulated. Solid bar indicates APP/E3 mice and pattern bar indicates APOE3 mice.

The development of amyloid phenotype can potentially affect transcription factor binding to genomic sites and therefore gene expression levels. To test this, we compared the effect of ligand activated RXR in APOE3 mice expressing either endogenous mouse *App* (APOE3 mice) or human *APP* gene (APP/E3 mice). A notable difference between the response of APP/E3 and APOE3 mice to bexarotene treatment was the significantly lower number of differentially affected genes: at p < 0.05 cut-off we identified 216 differentially affected genes in APP/E3 vs 2751 genes in APOE3 mice (Fig. 4A,B). As shown on Fig. 4C, there was a significant similarity in the categories of genes up-regulated by bexarotene between APOE3 and APP/E3 as well as with already published data for APOE4 mice^22^, and APP/PS1 mice expressing mouse APOE^24^. For example, top GO functional categories identified in up-regulated genes in APP/E3 mice included “neuron differentiation”, “vasculature development”, “neuron development”. Common genes up-regulated in both APOE3 and APP/E3 were: *Dlk1* (delta-like homologue 1)^30^, *Vgat/Slc6a12* (GABAergic vesicular transporter), *Apod* (apolipoprotein D), *Prph* (peripherin); *Rdm1* (involved in RNA-directed DNA methylation) among others. The up-regulation of *Dlk1*, an imprinted gene that acts as an atypical NOTCH ligand and regulates postnatal neurogenesis^30^,^31^ in both data sets confirms our previous finding that activated RXR affect NOTCH signaling^22^. We found that there was a significant difference in down-regulated categories (see Fig. 4D). In APP/E3 mice^31^ the top down-regulated were: “response to endogenous stimulus” (p < 10^−6^); “lipoprotein metabolic process” (p < 10^−4^); “acute phase response” (p < 10^−3^) and “inflammatory response” (p = 0.0016) reminiscent of the effect of bexarotene in APP/PS1 mice expressing mouse APOE (see also the results shown in Lefterov *et al*.^24^). In contrast, the top categories down-regulated by bexarotene in APOE3 mice were: “transcription” (p < 10^−9^), “mRNA metabolic process” (p < 10^−15^); “chromosome organization” and “chromatin modifications” (p < 10^−3^) (Fig. 4C). Of particular interest in terms of AD, because of their role in amyloid deposition and aging, were some genes up-regulated in APP/E3 mice but not affected in APOE3 such as Klotho (*Kl*)^32^,^33^ and transthyretin (*Ttr*)^34^. Interestingly, *Ttr* was also found up-regulated by bexarotene in APP mice expressing mouse Apoe^24^. In contrast, some genes with a role in Aβ clearance and phagocytosis such as *Lrp1*^35^, *Abca7*^36^ and Clusterin (*Clu*)^37^ were up-regulated by bexarotene in APOE3 mice but not in APP/E3 (Fig. 4A,B). These results indicate that amyloid deposition affects the transcriptome in mice expressing human APP and potentially can affect RXR binding to genomic sites and response to RXR agonists.

### Bexarotene treatment maintains neurite complexity and prevents loss of neurites in primary neurons exposed to Aβ

**Figure 5:**
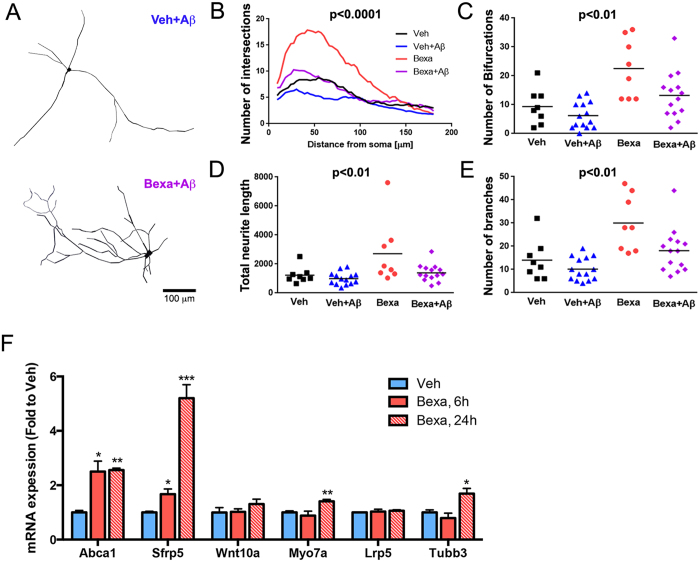
Bexarotene treatment maintains dendrite complexity and prevents loss of neurites in primary neurons exposed to Aβ. Mouse primary cortical neurons were infected with GFP expressing lentivirus at DIV0. DIV14 cells were pre-treated with 1 μM bexarotene or vehicle for 24 hours followed by 0.5 μM Aβ_42_ for 24 hours. (**A**) Digital reconstructed images of Aβ or Bexa followed by Aβ treated cells, respectively; (**B**) Number of Intersections. Statistics is by two-way repeated measures ANOVA with number of intersections as an independent variable, treatment group as a factor, and distance from the soma as the repeated-measure factor. There is an interaction F_(171,2280)_ = 6.23, p < 0.0001. Total neurite length **(C)**, Number of Bifurcations **(D)** and Number of branches **(E)** were significantly increased in the neurons treated with bexarotene. Statistics for (**C–E**) is by one-way ANOVA. For each treatment group, images were taken from at least 4 wells and of more than 2 GFP+ neurons per well. (**F**) Primary neurons were treated for 6 or 24 hours with bexarotene and mRNA level for selected genes related to categories “neuron projections” and “Wnt signaling” were examined by qPCR. Values are mean ± SEM; Statistics is by *t*-test; n = 3, *p < 0.05, **p < 0.01 versus vehicle group.

Previously, we demonstrated that bexarotene-activated RXR enhanced neurite branching and complexity in mature neurons^22^. To determine the neuroprotective effects of bexarotene against Aβ, we used primary neuronal cultures established from cortices and hippocampi of E16 mouse embryos. Following Aβ treatment for 24 hours, the morphology of GFP neurons was digitally reconstructed from high resolution images using Neuromantic^22^,^38^. Bexarotene-treated neurons demonstrated significantly increased numbers of intersections (Fig. 5B) and branches (Fig. 5C). Aβ_42_-treatment impaired number of intersection, branches and bifurcations in primary neurons. In contrast, pre-treatment with bexarotene before the addition of Aβ_42_ prevented loss of neurites and maintained neuronal complexity (Fig. 5A). To determine bexarotene effect on mRNA expression in primary neurons, we selected few genes identified in our RNA-seq datasets as RXR targets and associated with Wnt signaling and microtubule dynamics that potentially can affect neurite complexity: secreted Frizzled-related protein 5 (*Sfrp5*), *Wnt10a, Lrp5, Myo7a* and tubulin beta 3 class III (*Tubb3*). As visible from Fig. 5F, bexarotene increased expression of *Abca1* demonstrating target engagement. We also noted a significant up-regulation of *Sfrp5* (more than 4-fold, p < 0.001) and a small but statistically significant up-regulation of *Myo7a* (p < 0.01) and Tubb3 (p < 0.05).

### Bexarotene inhibits Aβ_42_ induced disruption of actin cytoskeleton in N2a cells

**Figure 6:**
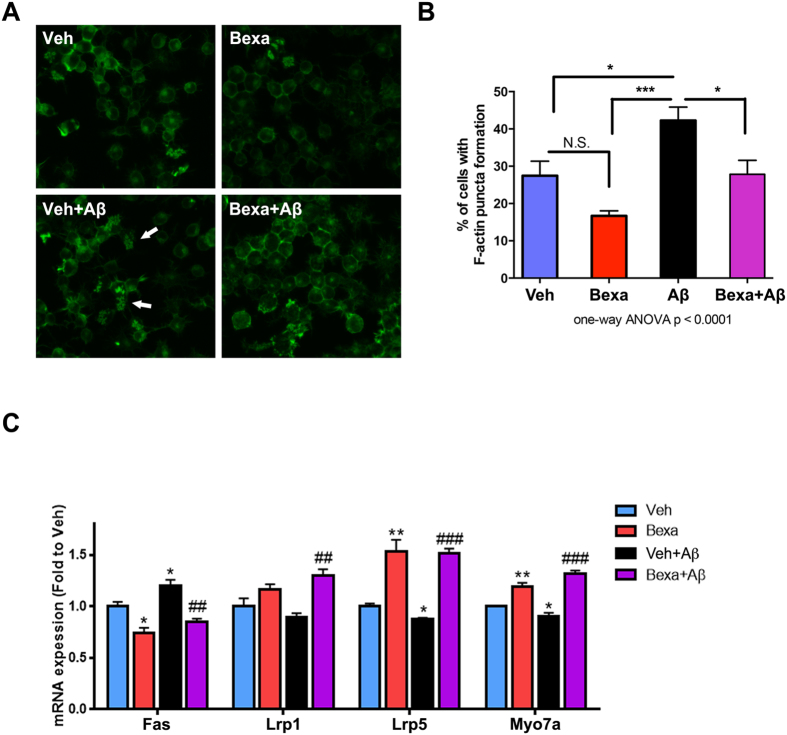
Bexarotene inhibits disruption of actin cytoskeleton caused by Aβ_42_ in N2a cells. N2a cells were treated with vehicle or 0.5 μM bexarotene for 24 hours and then treated with 0.5 μM Aβ_42_ for 24 hours. Cells were stained with alexa 488 phalloidin for visualizing F-actin puncta. (**A**) Representative images of phalloidin staining. The white arrows point intracellular accumulation of F-actin puncta. (**B**) The Quantification of F-actin puncta formation. The percentage of cells with F-actin puncta formation was calculated for each condition from merged DAPI and phalloidin images from 9 pictures per condition. Values are mean ± SEM and statistics is by one-way ANOVA followed by Tukey’s post-test; n = 9, *p < 0.05, ***p < 0.001. (**C**) N2a cells were treated as shown on A and B and mRNA level for selected RXR targets was examined by qPCR. Values are mean ± SEM; Statistics is by *t*-test; n = 3, *p < 0.05, **p < 0.01 versus vehicle group; ^##^p < 0.01, ^###^p < 0.001 versus Aβ group.

Previous studies reported that disruption of actin cytoskeleton and aggregation of actin was found in hippocampal and cortical neurons of post-mortem AD brains and cultured cell following chemical or physical stress^39^. The link between Rho family GTPases and Wnt signaling, members of common pathways identified by ChIP-seq and RNA-seq in mouse brain, affect cytoskeleton remodeling through cooperation^40^. Since these pathways regulate cytoskeleton organization, we examined bexarotene effects on F-actin puncta formation in Aβ_42_-treated N2a cells. First, we found that Aβ_42_ treatment increased F-actin puncta formation by 54% as compared to vehicle treated N2a cells (Fig. 6A,B). In contrast, bexarotene significantly inhibited F-actin aggregation as compared to vehicle or Aβ_42_ treated cells. Our data also demonstrate that bexarotene treatment prior to the addition of Aβ_42_ inhibited F-actin puncta formation (Fig. 6A,B). Next, using N2a cells, we tested the effect of bexarotene and Aβ_42_ on mRNA expression of several, already identified RXR targets (see Fig. 2) involved in Wnt signaling as well as neurite extensions. Consistent with the quantification of F-actin puncta in N2a cells, the expression of genes that affect cytoskeleton dynamics as well as neuron projections was decreased after Aβ_42_ treatment. In contrast, bexarotene treatment prior to Aβ application restored their expression (Fig. 6C). These data suggest that bexarotene inhibits disruption of actin cytoskeleton induced by toxic Aβ species.

## Discussion

Bexarotene-activated RXRs improve behavior deficits and increase Aβ clearance from brain interstitial fluid in mice expressing human APP^6^,^9^,^41^. Previously we reported bexarotene also increased the number of neuronal progenitors in brains of APOE4 and APOE3 mice and neuronal differentiation of mouse embryonic cells^22^. To compare if the effects of bexarotene on RXR cistrome and transcriptome in mouse cortex are affected by APOE isoform, we performed high throughput massive parallel sequencing using cortices from mice expressing human APOE3.

Using ChIP-seq, we found bexarotene promoted dynamic changes of RXR binding toward promoter region and affected genes associated with “transcription”, “chromatin remodeling” and “neuron projections” in APOE3 mice. To identify effects of APOE isoforms we applied IPA and compared functional categories identified by the cistrome analysis of APOE3 to those in APOE4 mice from our previous study^22^. We determined that highly enriched categories “nervous system development”, “axonal guidance”, “behavior” and “neurites extension” overlap in both genotypes. The analysis of the transcriptomes revealed similarity also in other functional categories of differentially affected genes related to neuronal differentiation and neuron projection that could be relevant to AD. These data were further validated in cell culture and *in vivo* using qPCR. Thus, the present findings demonstrate that APOE isoform by itself does not affect binding of RXR homodimers to genomic sites and consequently the effect on gene expression.

Several signaling pathways important for neuronal development and neurites extension, identified as significant in both APOE3 ChIP-seq and RNA-seq datasets also overlapped. Rho family GTPase was identified as highly enriched in ChIP-seq and up-regulated in RNA-seq dataset (Fig. 2B). Rho family GTPases are associated with cytoskeletal organization essential for neurite outgrowth and axon extension, as well as, for neurite branching and neuronal complexity^42^. They also play a role in regulating the dynamics of F-actin-rich cytoskeleton present in dendritic spines and are important for synaptic transmission^43^. Furthermore, abnormalities in cytoskeletal organization have been reported in many neurodegenerative disorders including AD^44^, and the disruption of neurite complexity and synaptic loss are closely related to neurodegenerative diseases^45^,^46^. Our data, shown on Fig. 6, demonstrate that oligomeric Aβ species disrupt actin cytoskeleton and bexarotene treatment counteracts this effect. Previously we have shown that bexarotene improves dendritic complexity and branching in primary neurons and in APOE3 and APOE4 mice^22^. In this study we demonstrate that bexarotene-activated RXR significantly reduced deleterious effects of oligomeric Aβ on neuronal processes, neurite length and branching in cultured primary neurons (Fig. 5).

Bexarotene treatment, as shown on Fig. 1D, increased RXR binding to *Wnt3* - a key member of Wnt signaling pathway, involved in nervous system development, differentiation and plasticity^47^,^48^. This finding is consistent with the pathway analysis of RNA-seq data (Fig. 2B). Several members of Wnt signaling pathway such as *Myo7a*^49^, *Lrp1*^50^, *Lrp5*^51^, *Wnt10a* and *Sfrp5* were identified by RNA-seq and further validated by qPCR in APOE3 cortex (Fig. 2D), N2a cells (Fig. 2) and primary neurons (Fig. 5F). SFRPs (secreted Frizzled-related proteins) are soluble ligands for the non-canonical Wnt signaling pathway involved in axon guidance^52^ and anti-inflammatory response^53^. *Lrp1* (LDL receptor related protein 1) is an endocytic receptor with a critical role in Aβ clearance^54^ possibly via interaction with Rho GTPases^35^. Lrp5 (LDL receptor-related protein 5) is Wnt signaling receptor expressed in endothelial cells, vascular smooth muscle cells and monocytes, reportedly involved in lipid internalization and motility of macrophages^55^. A recent study demonstrated that bexarotene ameliorated aging-related synapse loss depended on neuronal Lrp1^56^. The data from the current study suggest that bexarotene effects on *Lrp1* and *Lrp5* can account for ameliorating deleterious Aβ effects. As we demonstrate here, *Lrp5* expression is up-regulated in response to bexarotene treatment, apparently due to an increased RXR binding to its proximal promoter. In a further proof that direct up-regulation of *Lrp5* is associated with Aβ metabolism we show that bexarotene prevents the inhibitory effect of oligomeric Aβ on *Lrp5* expression (Fig. 6C).

Other pathways related to neuronal morphology and function identified as responsive to RXR treatment were CREB and CXCR4 signaling. The transcription factor CREB regulates genes involved in neuronal survival and plays an essential role for long-term synaptic plasticity^57^,^58^. Transcriptional up-regulation of several members of this pathway identified by RNA-seq, namely, *Akt1, Grin1, Grin2d* and *Grm4* was validated in APOE3 mice and *in vitro* (Fig. 2). *CXCR4* (CXC chemokine receptor-4) is expressed in neural stem cells and plays a role in the migration of the neural progenitors from the neurogenic zones to the sites of damage after brain injury^59^.

Since GO categories “neuron fate commitment”, “nervous system development” and “neurogenesis” were significantly affected in both APOE3 and APOE4 mice we examined if bexarotene induces changes in epigenome that coincide with the transition from pluripotent cells to neuronal progenitors. We performed ChIP-seq and mapped the enrichment of two histone marks - H3K4me3, associated with active promoters, and H3K27me3 - a mark for repressed promoters, using mouse embryonic stem cells. We demonstrate that bexarotene induces epigenetic changes in these cells consistent with increased neuronal differentiation and in correlation with an effect on transcription (Fig. 3).

The effect of amyloid deposition on the transcriptome in APP/E3 mice in response to bexarotene is an important finding. Evidence from studies using APP transgenic mice suggest amyloid deposition and other neuropathological features affect transcriptome changes that potentially could affect transcription factor binding^60^,^61^. We compared transcriptomes of APOE3 and APP/E3 mice and some of common genes that are involved in neuroprotective effects such as *Apod, Slc6a12, Prph* and *Rdm1* were identified in the up-regulated sets of genes in both genotypes. *Slc6a12* (solute carrier family 6, member 12, also called *vGat*) is a vesicular GABA transporter and a marker of GABAergic terminals. Recent report demonstrated that Aβ injected in the hippocampus decreased *vGluT1* (a marker of glutamatergic terminals) level without change in *vGAT*-immunopositive nerve terminals^62^. This suggests that the expression of *vGat* is less susceptible to the effect of amyloid deposition and is in agreement with our data. *Apod*, a gene which was found up-regulated by bexarotene in APP/PS1 mice^24^ has a neuroprotective, as well as, anti-oxidant, and anti-inflammatory role^63^. *Prph*, a neuron-specific TypeIII intermediate filament is involved in neurite outgrowth during development and axonal regeneration^64^. Consistent with our previously published data on bexarotene treated APP transgenic mice expressing mouse *Apoe*^24^, “immune system development” was one of the top GO functional categories identified in up-regulated gene sets in APP/E3 mice. Remarkably, however, most of the genes down-regulated by ligand activated RXR in those mice (Fig. 4), compared to APOE3 animals, clustered in significant, yet distinct, BP categories, associated with immune system and inflammatory response. Among them were “positive regulation of immune system”, “acute inflammatory response”, “acute-phase response” and “response to endogenous stimulus”. Based on highly reproducible GWAS and numerous experimental studies, it is now firmly established that microglia, immune and inflammatory reactions in CNS play substantial role in AD pathogenesis. While it is not entirely clear if AD genetic predisposition is primarily associated with immune function as suggested^60^, microglia are the non-neuronal CNS cells most intimately associated with AD pathological changes in brain^65^. Therefore, we interpret the differential response to bexarotene in APP/E3 and APOE3 mice as a response of complex, mixed macrophage phenotypes formed by clustered regulatory and metabolic pathways^66^,^67^. The data and validation assays presented here suggest that increased inflammatory reactions in APP mice expressing human APOE isoforms can be pharmacologically mitigated at the level of transcription factor binding and ultimately by modulating transcription efficiency of genes that participate in a number of immune and inflammatory pathways, and therefore provide one possible mechanism to establish therapeutic targets in human disease with strong genetic predisposition.

## Materials and Methods

All reagents, chemicals and standard additives, unless otherwise stated were purchased from Sigma Aldrich (St Louis, MO), or Thermo Fisher (Pittsburgh, PA).

### Mice

All animal experiments were approved by the University of Pittsburgh Institutional Animal Care and Use Committee. All procedures were carried out in accordance with the approved guidelines. Mice with APOE3^+/+^ targeted replacement (referred to as APOE3) were purchased from Taconic on C57BL/6 background and bred in house on the same background. APOE3 mice litters were used at 6 months of age. APP/PS1ΔE9 mice were bred to human APOE3^+/+^ targeted replacement mice to generate APP/PS1ΔE9/APOE3^+/+^ (referred to as APP/E3) mice expressing only human APOE3. Animals were randomly assigned to either bexarotene (100 mg/kg/day; oral gavage; Targretin, Eisai Inc., WoodCliff Lake, NJ) or vehicle (0.2 mg/kg glycerol) treatment groups.

### Processing of mouse brain tissue

Mice were anesthetized with Avertin (250 mg/kg of body weight, i.p.) and perfused transcardially with 25 ml of cold 0.1 M PBS, pH 7.4^68^. Brains were rapidly removed and divided into hemispheres. For RNA isolation, the brains were dissected and snap frozen on dry ice.

### Cell culture and treatment

Mouse Embryonic Stem (ES) cells, line R1 (ATCC, Manassas, VA) were grown on gelatin (0.1%) coated T75 flasks in standard ES cells culture medium composed of GMEM supplemented with 10% of ES cells qualified FBS, L-Glutamine 2 mM, non-essential amino acids (0.1 mM), 2-mercaptoethanol (0.1 mM), sodium pyruvate (1 mM), antibiotics and 100 U/ml LIF (mouse Leukemia inhibitory factor) (Life Technologies, Grand Island, NY). Treatment of cell cultures to induce neuronal differentiation of ES cells was as previously described^22^. Briefly, ES cells were plated in petri dishes in medium without LIF to initiate the formation of Embryoid Bodies (EBs). EBs were treated with 5 μM bexarotene dissolved in DMSO, or DMSO only for 4 days. Four days after treatment, EBs were plated on poly-L-ornithine/laminin-coated plates in N2B27 medium and differentiated into neurons. Cells were collected at 24 hours, 4 days and 10 days after the treatment for RNA isolation and collected 4 days after the treatment for Chromatin Immunoprecipitation. Mouse neuroblastoma N2a cells were cultured in DMEM/F12 supplemented with 10% FBS, antibiotics (Life Technologies, Grand Island, NY). For validation of bexarotene effect, N2a cells were treated with 1 μM bexarotene and RNA was isolated 24 and 48 hours after the treatment.

### Chromatin immunoprecipitation and sequencing (ChIP-seq)

Brains from APOE3 mice treated with bexarotene for 10 days and ES cells treated with bexarotene for 4 days were used for ChIP-qPCR and ChIP-seq. Brain lysates were sonicated with 3 pulses of 15 sec at amplitude 30, a 120 sec pause and 3 pulses of 15 sec at amplitude 40 using a Model 705 Sonic Dismembrator (Fisher Scientific, Pittsburgh, PA), to obtain fragments of 200–600 bp. For immunoprecipitation, we used a rabbit polyclonal anti-RXR (ΔN 197, #sc-774, Santa Cruz, CA), rabbit polyclonal anti-H3K4me3 (#CS200580, Millipore, CA) and rabbit-polyclonal anti-H3K27me3 (#07-449, Millipore, CA). For ChIP validation we performed ChIP-qPCR with mouse Glucagon (*Gcg*) as a negative control and determined Fold enrichment normalized to % input of negative control (target gene % input/negative control % input). ChIP libraries were generated using TruSeq ChIP Sample Prep Kit (Illumina, San Diego, CA) following manufacturer’s protocols. For each steps, samples were purified by AMPure XP beads (Beckman Coulter, Brea, CA). Adapter-ligated samples were separated on a 2% agarose gel to obtain 250–300 bp size-range of DNA fragment to remove unligated adapters. The libraries were validated by Agilent Technologies 2100 Bioanalyzer to check the size, purity, and concentration of the sample before the sequencing, and sequencing performed on Illumina HiSeq2000 instrument at the Functional Genomics Core, UPenn, Philadelphia (http://fgc.genomics.upenn.edu/). We used Bowtie2 (http://bowtie-bio.sourceforge.net/bowtie2) to align sequencing reads to the mouse genome (mm9 release) and Homer tools (http://biowhat.ucsd.edu) for peak calling, annotation and further processing of BAM/BED files.

### RNA isolation, qPCR and sequencing

RNA was isolated from brain and cultured cells and purified using RNeasy mini kit and QiaShredder (Qiagen, Valencia, CA) according to the manufacturer’s recommendations. The quality control of all RNA samples was performed on a 2100 Bioanalyzer instrument and samples with RIN >8 were further used for qPCR and library construction using mRNA Library Prep Reagent Set (Illumina, San Diego, CA). Libraries were generated by PCR enrichment including incorporation of barcodes to enable multiplexing. The libraries, 5 or 6 per condition (6 bexarotene and 5 vehicle treated males) were sequenced on Illumina HiSeq2000. Sequencing reads were aligned to the mouse genome (mm9 release) using Subread and differential expression analysis performed by edgeR^69^. For qPCR, first strand cDNA was synthesized from 1 μg of total RNA using EcoDry™ Premix, Random Hexamers (Clontech, Mountain View, CA). qPCR was performed using TaqMan^®^ Universal Master Mix II (Life Technologies, Grand Island, NY). Transcript levels were normalized to *Gapdh* and amplification plots were analyzed by comparative ΔΔCt method.

### Functional Pathway analysis and Comparative analysis

To identify biological process changes and the most relevant pathways related to the effects of RXR and epigenetic changes, we performed functional annotation clustering using the Database for Annotation, Visualization and Integrated Discovery (DAVID, https://david.ncifcrf.gov/)^26^ and Ingenuity Pathway Analysis (IPA^®^, QIAGEN, Redwood City, www.qiagen.com/ingenuity). For comparative analysis and graphics, R was used (https://www.r-project.org/): proportional Venn diagrams were generated using package VennDiagram; clusters that were generated by IPA were visualized as heatmaps using package gplots. Volcano and scattered plots were generated for visualizing differential gene expression.

### Preparation of amyloid beta oligomers

Aβ_42_ were synthesized at Keck’s facility (Yale University) and stock solutions of synthetic Aβ_42_ were disaggregated according to the method described previously^24^,^70^. In brief, 1 mg Aβ was dis-aggregated first with trifluoroacetic acid and hexafluoro-2-propanol, followed by removal of the solvents. The aqueous dissolution of the resulting disaggregated peptide film was followed by high speed centrifugation (100,000 × *g*) for 1 h to remove trace aggregates. This protocol yields mostly monomeric Aβ as determined by Western blotting (not shown). Aβ aggregation reactions were performed in serum-free medium at room temperature (20 °C) without shaking.

### Phalloidin staining

N2a cells were plated on 18 mm circular coverslips coated with 0.01% poly-L-lysine. The cells were treated with vehicle or 0.5 μM bexarotene for 24 hours and then treated with 0.5 μM Aβ_42_ for 24 hours. Cells were fixed with 3.7% paraformaldehyde for 10 min at room temperature and stained with Alexa 488 phalloidin (Life Technologies, Grand Island, NY) for visualizing F-actin puncta. DAPI was used for counterstaining and multi-channel GFP-DAPI images (1392 × 1040 pixels) captured with Nikon 90i microscope using 20× objective.

### Primary embryonic mouse cortical neuronal cultures and treatment

Primary mouse cortical neuronal cultures were prepared from E16 embryos. Single cell suspension was used and cells plated at a density of 10^5^ cells per cm^2^ on 18 mm circular coverslips coated with 0.01% poly-L-lysine in Neurobasal medium containing antibiotics, L-glutamine, and B27 supplement. Cultures were maintained by replacing 50% of the conditioned medium with fresh medium, every 2 days. At the day of dissection (DIV0), cells were infected with GFP-Lentiviral particles (pLVX-IRES-ZsGreen1 Vector; Clontech, Mountain View, CA) at 0.2 multiplicity of infection. The moderate efficiency of this infection technique allows an easy identification of the processes associated with single neurons. At DIV14, infected neurons were pre-treated with 1 μM bexarotene or vehicle for 24 hours followed by 0.5 μM Aβ_42_ for 24 hours. Six days later (at DIV21), the neurons were fixed with 4% paraformaldehyde for 20 min at room temperature, counterstained with DAPI, and multi-channel GFP-DAPI images (1392 × 1040 pixels) captured with Nikon 90i microscope using 20× objective.

### Neuronal reconstruction and morphological analysis

For each treatment group, at least 4 wells and no less than 2 GFP+ neurons per well were imaged. DAPI staining was used to verify that only single neurons, but not cell clusters, were selected for reconstruction. The GFP channel of each captured image was then exported as a TIF file, and individual neurons were semi-automatically reconstructed using Neuromantic (Myatt, 2012). Neuronal reconstructions, saved as SWC files, were used for subsequent morphometric analyses. Sholl analysis was additionally performed using the Trees Toolbox (Cuntz, 2011) for Matlab (Mathworks).

### Statistics

All results are reported as means ± S.E.M. All statistical analyses (if not otherwise mentioned in the text) were performed in GraphPad Prism, version 6.0 (LA Jolla, CA) and differences considered significant where p < 0.05.

### Dataset

The datasets in a proper format will be uploaded to a publicly available repository, and is directly available from Koldamova&Lefterov Laboratory.

## Additional Information

**How to cite this article**: Nam, K. N. *et al*. RXR controlled regulatory networks identified in mouse brain counteract deleterious effects of Aβ oligomers. *Sci. Rep.* **6**, 24048; doi: 10.1038/srep24048 (2016).
